# South West Radiologists Association

**Published:** 1992-03

**Authors:** 


### South West Radiologists Association

### Meeting at Exeter, June 1991

### URODYNAMICS FOR THE UNINITIATED

**Paper presented by Dr. Charles E. Corney at meeting on 28th June 1991 of the South West Radiologists Association at the Royal Devon and Exeter Hospital, Exeter**

A MORI poll earlier this year indicated that nearly 4 million people in the United Kingdom are incontinent. These patients have yet to appear in large numbers for investigation and treatment mainly because of reticence on their part, but also because of failure of doctors in general to understand that incontinence can be investigated and treated successfully in a majority of cases. Also some gynaecologists and surgeons have had difficulty in understanding the seemingly complicated technique of urodynamic investigation. A simple investigative bladder pressure flow technique is described — express in simpler scientific terms. If the result is equivocal then X-ray imaging, urethral pressure monitoring and electromygraphy of the distal sphincter can be added to reach the definitive cause or diagnosis. Then and only then treatment can be commenced. Treatment of symptoms (which are often diagnostically unreliable) without preliminary urodynamic investigations is to be deprecated as failure is very likely.

Details of the diagnosis, drug and surgical treatment of common conditions such as stress incontinence, destrusor instability, neuropathic bladder, outlfow obstruction and iatrogenic effect on the bladder and urethra from incidental drug therapy for disease of other parts of the body, and from previous TUR and repair operations are described. Perhaps there should be a national screening programme similar to Forrest breast screening, to highlight the problem of incontinence allowing earlier treatment.

### DOPPLER ULTRASOUND IN THE ABDOMEN — IS IT WORTH THE EFFORT?

**Presented by Dr. C. Hamilton-Wood at the South West Radiologists Association meeting on 28th June 1991**

The basic physics of doppler ultrasound was discussed, and various examples were taken and applications of doppler ultrasound were described, providing evidence to justify the use of doppler ultrasound scanning in several areas of abdominal scanning.

Sorting out difficult areas of complex normal anatomy, vascular complications of various pathologies, applications in urology, obstetrics and gynaecology, abdominal trauma and transplantation were all described and examples cited.

Particular emphasis was placed on the use of doppler ultrasound in renal transplantation, and a description of the ultrasonic appearances of transplant rejection in various degrees of severity were discussed.

The conclusion was drawn that doppler ultrasound may provide unique information to help the abdominal ultrasonographer, and not infrequently provides information which otherwise could be obtained without this facility, but very much more quickly.

### MAGNETIC RESONANCE IMAGING — A TARGETTED APPROACH

**Paper presented by Dr. W. Vennart at the South West Radiologists Association Meeting on 28th June 1991 at the Royal Devon & Exeter Hospital (Wonford), Exeter, Devon.**

Magnetic Resonance Imaging (MRI) systems involve a high capital cost. For this reason there are only a few systems (compared with X-ray CT) installed in the United Kingdom for clinical use and not many centres are carrying out fundamental research into MRI. The MRI group at the University of Exeter have been involved with the development of low cost instrumentation, and, in particular, its application to arthritis research. High resolution images (200 x 100m) in-plan resolution of the finger joints have been generated using an 18cm bore superconducting magnet system. These images demonstrate the various zones of articular cartilage, namely a superficial layer, an area giving an intense water signal and finally calcified cartilage attached to bone surfaces. Another system is devoted to imaging the wrist and forearm; this imager is used for monitoring changes in the wrist joints as arthritis develops and for making fundamental measurements on muscle tissue, e.g. diffusion coefficients. A completely new development is the use of actively-screened magnetic field gradient sets in conjunction with a 560mm bore magnet for imaging the knee and head. This system is under development but already demonstrates the potential for brain imaging.

The majority of imaging worldwide using MRI is centred on the brain and musculoskeletal system. It is clear, therefore, that the possibility of targetted MRI systems both for clinical imaging and research, hold great potential.

### PUT YOUR NEEDLE WHERE THE MONEY IS

**Presented by Dr. C. R. Bayliss at the South West Radiologists Association meeting on 28th June 1991.**

Vascular imaging has changed and developed remarkably over the last decade. The advent of digital Subtraction Angiography and smaller catheter/needle systems has meant that suitable patients can now be investigated on an outpatient or day case basis.

In addition, the Vascular Radiologist now has therapeutic tools at his disposal — particularly angioplasty and thrombolysis. It is therefore suggested that patients should no longer have a 'diagnostic' study first, followed at a later date by an interventional procedure. By 'putting your needle where the money is' it usually proves possible to do a single combined diagnostic and therapeutic study.

Various examples of stenoses, occlusions and acute thromboses in the aortoiliac and more distal segments, and their successful treatment, where shown and some of the problems and complications encountered were discussed.

